# Implementação de Programas de Melhoria de Qualidade Assistencial

**DOI:** 10.36660/abc.20200679

**Published:** 2020-07-28

**Authors:** Felix J. A. Ramires

**Affiliations:** Universidade de São Paulo Instituto do Coração São PauloSP Brasil Universidade de São Paulo Instituto do Coração , São Paulo , SP – Brasil

**Keywords:** Doenças Cardiovasculares/mortalidade, Prevalência, Guias como Assunto, Envelhecimento, Fatores de Risco, Ocupação de Leitos, Tratamento Farmacológico, Melhoria de Qualidade, Redução de Custos

A incidência e a prevalência das doenças cardiovasculares crescem em todo o mundo. ^1 - 3^ Tal fato se dá, em parte, ao envelhecimento da população e ao acúmulo de fatores de risco associados a essas doenças. No Brasil, a expectativa de vida da população em 2019 foi estimada em 76,6 anos em uma população de 211.652.819 pessoas, avaliada pelo IBGE. Neste mesmo ano, foram registradas aproximadamente 12.168.390 internações no Sistema Único de Saúde (SUS) — Ministério da Saúde — sendo aproximadamente 1.200.000 por doenças cardiovasculares (10% do total). ^4^ Além disso, a mortalidade relacionada às doenças cardiovasculares mantém-se elevada, por volta de 29% das causas de óbito anuais. ^5^ Portanto, observamos o grande impacto dessas doenças no que se refere à ocupação de leitos hospitalares e a mortalidade no Brasil. Neste cenário, programas de gerenciamento de doenças crônicas têm demonstrado que o acompanhamento monitorado e multidisciplinar melhora a adesão ao tratamento farmacológico e não farmacológico determina otimização da terapêutica, diminui o número de hospitalizações relacionadas diretamente à doença promovendo melhora importante na qualidade de vida e redução dos custos hospitalares. ^6 - 8^ Registros brasileiros têm evidenciado a aderência às diretrizes médicas extremamente baixas. ^9^ As razões para um baixo desempenho na implementação de diretrizes clínicas incluem barreiras relacionadas ao próprio sistema de saúde, ao empenho e ao aprimoramento médico, ao envolvimento multidisciplinar e ao envolvimento do próprio paciente no cuidado. Alguns estudos sugerem que por volta de 30%–40% dos pacientes não recebem atendimento de acordo com a evidência científica atual, enquanto 20% ou mais dos cuidados prestados não são necessários ou potencialmente prejudiciais. As estratégias desenvolvidas para otimizar a aderência às diretrizes atuais têm demonstrado sucesso no manejo desses pacientes. Esse gerenciamento de doenças crônicas, voltado para a qualidade do atendimento baseada em evidências, pode promover redução de eventos clínicos. Alguns estudos, de gerenciamento e melhoria da qualidade, demonstraram redução na taxa de readmissão hospitalar em 30 dias com a adoção dessas medidas. ^10 , 11^ Outros modelos, no monitoramento de desempenho associado a estratégias de implementação de diretrizes, promovem a melhora do cuidado e redução de desfechos. O estímulo ao cuidado e medidas de qualidade são heterogenias em diferentes regiões e entre instituições brasileiras, resultando em desfechos também extremamente variados. A associação de medidas de desempenho, detecção de oportunidades de melhorias e desenvolvimento de estratégias para o aumento da adesão às boas práticas em saúde são fundamentais para otimização de resultados.

Neste cenário, o desenvolvimento de protocolos e estudos nacionais de gerenciamento de doenças cardiovasculares, com foco na implementação de modelos e ferramentas de melhoria na qualidade do atendimento e na implementação da adesão às melhores práticas, tem um fundamental papel na avaliação da exequibilidade e do resultado. ^12^
