# Supplementary material for: Implementação de Programas de Melhoria de Qualidade Assistencial
Source: Arq Bras Cardiol. 2020 Jul 28;115(1):100–1. [Article in Portuguese] doi: 10.36660/abc.20200679 (PMC8384331; doi:10.36660/abc.20200679)
Supplement: Translation

# Translation: Implementation of Healthcare Quality Improvement Programs

**Authors:** Felix J. A. Ramires

**Affiliations:** Brazil Universidade de São Paulo Instituto do Coração, São Paulo, SP – Brazil

**Keywords:** Cardiovascular Diseases/mortality, Prevalence, Guidelines as Topic, Aging, Risk Factors, Bed Occupancy, Drug Therapy, Quality Improvement, Cost Savings

The incidence and prevalence of cardiovascular disease are increasing worldwide.^1 - 3^ This is partly due to population aging and the accumulation of risk factors associated with these diseases. In Brazil, the life expectancy of the population in 2019 was estimated at 76.6 years in a population of 211,652,819 people, assessed by IBGE. In the same year, approximately 12,168,390 hospitalizations were registered in the public healthcare system (in Brazil, SUS) — Ministry of Health — with approximately 1,200,000 due to cardiovascular diseases (10% of the total).^4^ In addition, the mortality related to cardiovascular diseases remains high, around 29% of the annual causes of death.^5^ Therefore, we observe the great impact of these diseases with regard to the occupation of hospital beds and mortality in Brazil. In this scenario, chronic disease management programs have shown that monitored and multidisciplinary monitoring improves adherence to pharmacological and non-pharmacological treatment determines optimization of therapy, decreases the number of hospitalizations directly related to the disease, promoting an important improvement in the quality of life and reduction of hospital costs.^6 - 8^ Brazilian records have shown adherence to extremely low medical guidelines.^9^ The reasons for poor performance in implementing clinical guidelines include barriers related to the health system itself, medical commitment and improvement, multidisciplinary involvement and the patient’s own involvement in healthcare. Some studies suggest that about 30%–40% of patients do not receive healthcare according to the current scientific evidence, while 20% or more of the healthcare provided are not necessary or potentially harmful. The strategies developed to optimize adherence to current guidelines have demonstrated success in the management of these patients. This chronic disease management, focused on the quality of evidence-based care, can promote the reduction of clinical events. Some studies on management and quality improvement have shown reduced rates of hospital readmission in 30 days with the adoption of these measures.^10 , 11^ Other models, in performance monitoring associated with strategies for implementing guidelines, promote the improvement of healthcare and reduction of outcomes. The encouragement of healthcare and quality measures are heterogeneous in different regions and among Brazilian institutions, resulting in extremely varied outcomes. The association of performance measures, detection of opportunities for improvement and development of strategies to increase adherence to good health practices are fundamental to optimize results.

In this scenario, the development of national protocols and studies on the management of cardiovascular diseases, focusing on the implementation of models and tools to improve the quality of healthcare and implementation of adherence to best practices, has a fundamental role in assessing feasibility and results.^12^
